# Opportunistic parasitic infections in patients with human immunodeficiency virus/acquired immunodeficiency syndrome: A review

**DOI:** 10.14202/vetworld.2020.716-725

**Published:** 2020-04-17

**Authors:** D. A. Laksemi, L. T. Suwanti, M. Mufasirin, K. Suastika, M. Sudarmaja

**Affiliations:** 1 Department of Parasitology, Faculty of Medicine, Udayana University, Bali, Indonesia; 2 Department of Parasitology, Faculty of Veterinary Medicine, Universitas Airlangga, Surabaya, East Java, Indonesia; 3 Institute of Tropical Disease, Universitas Airlangga, Surabaya, East Java, Indonesia; 4 Department of Internal Medicine, Faculty of Medicine, Udayana University, Bali, Indonesia

**Keywords:** epidemic, immune suppression, opportunistic infection, protozoa

## Abstract

The number of human immunodeficiency virus (HIV) cases increases annually, and Indonesia has become the country with the fastest HIV/acquired immunodeficiency syndrome (AIDS) epidemic spread among the five Southeast Asian countries. Indonesia entered the critical phase of HIV/AIDS infections after 5 out of the 33 provinces, namely, Papua, Jakarta, Bali, West Java, and East Java, reported HIV/AIDS epidemic since 2004. In AIDS pathophysiology and immune-suppression are severe, thus, opportunistic intestinal parasitic infections that cause diarrhea in HIV infection may be fatal. Several studies have suggested that *Cryptosporidium parvum*, *Isospora belli*, and *Blastocystis hominis* are the most common intestinal protozoan parasites categorized as AIDS associated illness. Diarrhea caused by parasites is considerably suspected in the cases of chronic and persistent diarrhea in adults, in an era of increasing HIV/AIDS cases nowadays. The present review highlights the current advances in etiologic agents of HIV/AIDS opportunistic infections among countries, epidemiology and prevalence, lifecycle, risk factors, examination methods, and treatment.

## Introduction

Opportunistic parasitic infections are infections of parasite species that are mild or asymptomatic in immunocompetent people; however, in immunocompromised people they become fatal [1]. Opportunistic parasitic infections, including worms and protozoa, are neglected tropical diseases that are targeted by the sustainable development goals to be eliminated by 2030 [2,3].

Human immunodeficiency virus (HIV) infection causes acquired immunodeficiency syndrome (AIDS), which is a complicated disease in humans, and suppresses the immune system [4,5]. Opportunistic parasitic infections are one of the most common health problems in patients with HIV/AIDS. The presence of opportunistic parasitic infections in HIV sufferers indicate that they are in the phase of AIDS. Most of these infections are severe, and often contribute to the death of infected individuals [4-7]. Thus, early detection and treatment need to be carried out to ensure adequate management [8-10].

The first case of HIV/AIDS in Indonesia was reported in Bali in 1987, while in Sulawesi it was reported in 2012 [11]. It is estimated that every minute, one Indonesian is infected with HIV/AIDS. The age group most affected is the productive age, i.e., 20-39years, with a case fatality rate of 18.7% [11].

Among the opportunistic infections, parasites are the most common causative pathogens that affect the morbidity and mortality of patients infected with HIV/AIDS. Parasites can trigger an immune response to infect the respiratory tract, digestive tract, blood, brain, and other organs. The parasitic species that most often cause the opportunistic infections in the human body are *Toxoplasma gondii*, *Cryptosporidium parvum*, *Isospora belli*, *Cyclospora cayetanensis*, *Microsporidia*, *Cryptococcus neoformans*, *Pneumocystis carinii* or *jiroveci*, and *Entamoeba* [12-14].

Among the parasitic opportunistic infections (POIs), cryptosporidiosis, isosporiasis, and microsporidiosis are the main enteric POIs, and toxoplasmosis and leishmaniasis are the main systemic POIs reported in HIV-infected patients [2,4,6]. Another study described the opportunistic parasites *C. parvum, Cyclospora cayetanensis, I. belli, and Microsporidia* spp. as a common feature in HIV/AIDS patients, especially when the CD4+ T cell counts fall below 200cells/μL [4].

Opportunistic infection has been categorized according to CD4+ counts as follows: *Mycobacterium tuberculosis* was found in all CD4+ counts; Coccidioidomycosis was in CD4+ counts <250cells/mm^3^*; Pneumocystis jiroveci* pneumonia and mucocutaneous candidiasis were in CD4+ counts <200cells/mm^3^; *Histoplasma capsulatum* was in CD4+ counts <150cells/mm^3^; *C. neoformans*, Cryptosporidiosis, Herpes simplex viruses, Microsporidiosis were in CD4+ counts <100cells/mm^3^; and *C. neoformans*, Cryptosporidiosis, and Cytomegalovirus, *T. gondii*, and Bartonellosis were in CD4+ count <50cells/mm^3^ [15].

UNAIDS data on the current HIV/AIDS situation have reported that globally 1.7 million people became newly infected with HIV by the end of 2018, 37.9 million people globally were living with HIV by the end of 2018, and almost 1 million people died from AIDS-related illnesses. Opportunistic parasitic infection is a major cause of morbidity and mortality worldwide; most of them are emerging diseases [11].

An understanding of parasites that cause opportunistic infections in HIV/AIDS is required, especially in the decades, where HIV has caused a huge burden on global wealth and health. Thus, early detection and prompt treatment can be established to reduce deaths due to HIV/AIDS.

This reivew presents an overview of the etiologic agents of opportunistic infections among countries, epidemiology and prevalence, lifecycle, risk factors, examination methods, and treatments.

## Clinically Relevant Intestinal Parasitic Infections

Parasitic infections in the digestive tract remain a burden for people infected with HIV, even in the era of the use of antiretroviral therapy (ART) [16]. CD4+ count in HIV patients are risk factors for opportunistic parasitic infections with manifestations of diarrhea [4].

In AIDS with severe immunosuppression, commensal intestinal parasites become opportunistic, causing fatal prognostic diarrheal diseases in HIV patients. There are various species of parasites that cause diarrhea in HIV patients, such as *C. parvum*, *Giardia lamblia*, *Microsporidia*, and *I. belli* [13,14]. Some studies have reported that the most common intestinal parasitic species found in diarrhea experienced by HIV patients are *Blastocyst* spp., *C. parvum*, *Microsporidia*, *S. stercoralis*, and O*. viverrini* [17]. There are also parasites known as classical opportunistic agents, which are *C. parvum*, *I. belli*, *C. cayetanensis*, and *Microsporidia* [17].

A study reported that the introduction of the highly active anti-retroviral therapy regimen as the main treatment for HIV has led to the occurrence of immune reconstitution inflammatory syndrome in opportunistic parasitic infections such as *C. parvum* [12].

## Risk Factors for Opportunistic Parasitic Infection

Risk factors for HIV/AIDS are man [7,18,19], unemployment, living in urban area, and marriage [19]. However, other studies have found that women, housewives, and trading are risk factors for HIV/AIDS [19].

In HIV/AIDS patients, the rate of a particular intestinal parasitic infection depends on the endemicity of the parasite in the community [7]. Intestinal parasites are widely distributed partly due to the low level of environmental and personal hygiene, fecal contamination of food and drinking water, and poor housing facilities [4]. Assessing current CD4+ cell count helps identify the status of intestinal parasite infection among HIV patients [20].

Risk factors associated with a higher prevalence of opportunistic parasitic infection among HIV patients were low CD4+ counts, persistent diarrhea, poor living conditions, and poor nutrition [20]. Another research reported ART status, CD4+ T-cell count, diarrhea, work status, and access to a toilet as the risk factors associated with a higher prevalence of intestinal parasites among HIV patients [19].

The association between the CD4+ T-cell count and intestinal protozoa infection has also been reported in the previous studies from Ethiopia, India, and Malaysia [4,18]. Stage of HIV/AIDS, CD4+ count, ART adherence, and hemoglobin level has also been reported as risk factors for opportunistic parasitic infections. CD4+ count <200cells/μL poses the greatest risk for opportunistic infection in HIV/AIDS patients [19].

Risk factors for *C. cayetanensis* are consumption of raw fruits and vegetables, drinking untreated water, swimming in rivers, contact with soil or animals, agricultural work, and poor hygiene [21]. Risk factors for *C. parvum* infection are non-Hodgkin’s lymphoma, leukemia, lymphoproliferative disease, malnutrition, immunosuppressive drugs, cancers, and hemodialysis [22]. Risk factors for *I. belli* include lymphoblastic leukemia, adult T-cell leukemia, Hodgkin’s disease, non-Hodgkin’s lymphoma, lymphoproliferative disorders, renal transplant, and liver transplant [22].

Risk factors associated with *Microsporidia* infection include sexual relations between men, use of intravenous drugs, exposure to swampy water or irrigated areas, exposure to water with feces, and use of swimming pools and hot tubs, or contact with water [23]. Research shows that *Microsporidia* is often found in patients with malignancy and diabetes mellitus [22].

Intestinal parasitism during pregnancy may affect the health of pregnant women and their offspring [24]. However, only few studies have described the effects of intestinal parasitism on pregnancy outcomes [25]. Parasitic infection(s) during pregnancy have been associated with increased risk of pregnancy complications and adverse outcomes [25]. Low weight during pregnancy, poor fetal growth, low birth weight, and preterm birth are some of the consequences of intestinal parasitic infection during pregnancy [24].

One of the intestinal parasitic infections that had a significant consequence on pregnant women and their offspring was *Blastocystis hominis*, which recorded 15% (50/331) prevalence among pregnant women in Bogota, Colombia [24]. *C. parvum* and *I. belli* can be transmitted from human to human through anal-oral contact [26]. Another research stated that marital status was not implicated as a determinant of the occurrence of enteric protozoal infection among the study subjects [18].

### C. cayetanensis

*C. cayetanensis* was first discovered in the form of blue-green algae, or coccidian-like body, or cyanobacterium-like body. This form of *C. cayetanensis* is without organelle, but has a membrane, and is larger in size than *C. parvum*. Traced phylogenetically, *C. cayetanensis* is closely related to the genus *Eimeria* [27].

**Figure-1:**
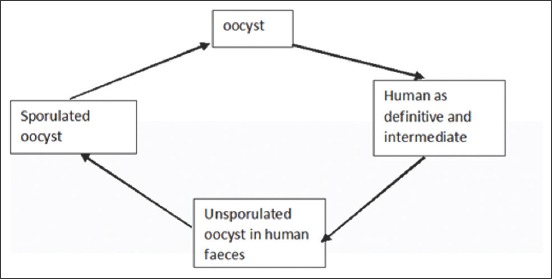
Life cycle of *Cyclospora cayetanensis*.

*C. cayetanensis* oocysts are found in raspberries and water sources that have the potential to cause epidemics [27]. In the lifecycle of *C. cayetanensis*, oocysts must be in the form of sporocysts outside the human body before becoming infective [28]. The lifecycle of *C. cayetanensis* (Figure-1) starts from the release of oocysts in feces. After about 7days of oocyst sporulation, it becomes an infective form. The sporocysts release sporozoites that infect the duodenum and jejunum, and then the sporozoites undergo asexual and sexual multiplication to produce oocysts that are non-sporulating in the host environment [27].

Patients suspected of having *C. cayetanensis* infection may present with symptoms of flu-like illness, diarrhea, anorexia, nausea, flatulence, fatigue, abdominal cramps, and mild fever accompanied by weight loss. Severe symptoms include ventricular dysrhythmia, biliary disease, Reiter syndrome, Guillain-Barre syndrome, ocular inflammation, sterile urethritis, and oligoarthritis [27].

Envirochek capsules and Hannifin filters can be used to get *C. cayetanensis* oocysts from water sources, while getting *C. cayetanensis* in the food can be performed through lectin (agglutination of wheat germ) coated with paramagnetic beads [29].

Parasitological diagnosis of *C. cayetanensis* is confirmed by finding spherical forms containing morula (oocyst) in the wet mount and stained preparations, including modified acid-fast stain, Giemsa, trichrome, Gram-chromotrope, and Kinyoun staining [27]. The method of fecal examination used for the diagnosis of opportunistic parasitic infections includes direct saline, iodine wet mount, formalin-ether sedimentation concentration, and modified Ziehl–Neelsen [30]. Molecular techniques using the PCR method of internally transcribed spacer 2 of DNA were performed to obtain a segment of 116bp from the sample [27].

The first choice of treatment for *C. cayetanensis* infection is trimethoprim-sulfamethoxazole (TMP-SMX) 25mg/kg body weight for 3days, other alternative treatments are ciprofloxacin, norfloxacin, azithromycin, tinidazole, nalidixic acid, diloxanide furoate, and quinacrine [27].

### C. parvum

*C. parvum* is an intracellular obligate parasite [31]. There are 31 species of *C. parvum* [32]. The parasite did not only cause diarrhea but it also manifested several symptoms in the lungs of HIV/AIDS patients [26].

Coccidian parasites (*Cryptosporidium* spp., *I. belli*, and *Cyclospora* spp.) are the most common enteric parasites in immunocompromised patients, which can cause severe, deadly diarrhea. The prevalence rate of cryptosporidiosis among diarrhea patients with HIV/AIDS is significantly higher than 10%. Immunocompromised diarrhea tends to be chronic and causes increased morbidity and mortality in these patients [33].

Cryptosporidiosis is a zoonotic disease; livestock, wild animals, pets, and rats may play an important role as the reservoir. It can be transmitted from human to human through anal-oral contact or from humans to animals through fecal-oral [26]. Besides HIV/AIDS, individuals with immunodeficiency conditions, such as those who have received chemotherapy, organ, or bone marrow transplants, and malnutrition and diabetes mellitus patients are also prone to infection by *C. parvum*. Other risk factors for *C. parvum* infection are non-Hodgkin’s lymphoma, leukemia, lymphoproliferative disease, immunosuppressive drugs, cancers, and hemodialysis [22].

**Figure-2:**
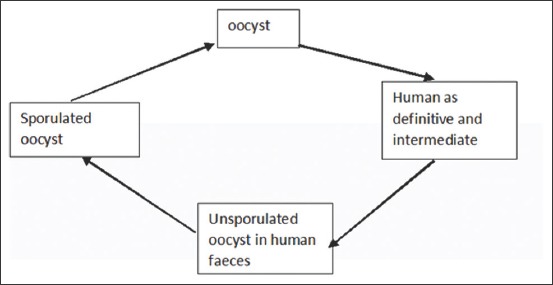
Life cycle of *Cryptosporidium* *parvum*.

The lifecycle of *C. parvum* (Figure-2) begins by ingestion of oocysts from contaminated water or food [34]. The parasite invades the intestinal epithelium, where it completes its lifecycle in the cytoplasm of the enterocyst. Unsporulated oocysts are excreted in feces and mature outside the host, where they develop into the infective sporulated oocysts [22]. *C. parvum* has a single host lifecycle that is asexual and sexual and occurs in the intestines of infected hosts [34].

Diarrhea caused by *C. parvum* in immunocompromised patients may be disseminated, thus causing increased mortality rate in HIV/AIDS [14]. *C. parvum* infection affects multiple organ functions, particularly the respiratory, pancreatic, and biliary tracts [35].

Microscopic examination is required to confirm the diagnosis of *C. parvum* infection. It can be performed by floatation, concentration, and Sheather’s sugar solution [36]. Further examination can be performed through immunofluorescence assay (IFA), antigen (immunoassay), and polymerase chain reaction (PCR) [35,37]. Various rapid diagnostic tests (RDT) are available including Immunocard STAT, RIDA check, Quick check, Giardia-crypto duo-strip, and RDT, which generally combine the examination of *C. parvum* with *G. lamblia* [38].

There are no commercially available human vaccines for *C. parvum*, although numerous candidate attenuated and DNA vaccines have been identified [35,37]. Oocysts of *C. parvum* have acid-fast (lipid-rich) walls, which are resistant to environmental insults and to gastrointestinal acids, proteases, and bile [37]. Prevention strategy must focus on proper hygiene and clean sanitary conditions to minimize *Cryptosporidium* outbreaks [35].

The pattern of opportunistic parasite species that is often found in people with HIV/AIDS in a country actually describes the opportunistic parasite species that exist in the country’s natural environment. This is due to suppression of the immune system in people with HIV/AIDS, making the individual vulnerable to infections of opportunistic parasites in their environment.

Many research had been reported different protozoal agents that caused diarrheal disease in HIV/AIDS. The opportunistic parasite infection pattern in each country is different (Table-1).

**Table-1:** Different protozoal agents in diarrheal disease based on geographical regions.

Parasite species	References	Published year	Country
*Cryptosporidium parvum, Cyclospora cayetanensis, Isospora belli* and Microsporidium	[1]	2016	Cameroon
*Cryptosporidium parvum, Isospora belli, Giardia lamblia, Entamoeba histolytica, Iodamoeba butschlii*	[3]	2015	India
*Cryptosporidium parvum, Isospora belli, Cyclospora, Microsporidia, Entamoeba histolytica*	[4]	2016	India
*Cryptosporidium parvum, Giardia lamblia, Leishmania tropica, Pneumocystis carinii*	[6]	2016	Egypt
*Cryptosporidium parvum, Entamoeba histolytica, Isospora belli, Taenia, Giardia lamblia*	[7]	2016	India
*Cryptosporidium* spp.,*Cystoisospora belli, Cyclospora cayetanensis, Giardia lamblia, Entamoeba histolytica, Blastocystis hominis, Microsporidia*	[11]	2015	India
*Blastocyst hominis, Cryptosporidium parvum, Microsporidia, Strongyloides stercoralis, Opisthorchis viverrini, Entamoeba dispar, Giardia lamblia*	[13]	2014	Laos
*Cryptosporidium parvum, Isospora belli, Blastocyst hominis*	[14]	2018	Ethiopia
*Cryptosporidium parvum, Isospora belli, Microsporidia, Cyclosporacayetanensis, Giardia lamblia*	[19]	2015	Ethiopia
*Cryptosporidium parvum, Enterocytozoon bieneusi, Giardia lamblia, Sarcocystis, Blastocyst hominis*	[20]	2013	Iran

### Microsporidia

*Microsporidia* is an intracellular obligate organism classified as eukaryotes because it has membranes, intra-cytoplasmic membrane systems, and chromosome separation in mitotic-spindles, and is closest to fungus because it contains chitin on the spore wall, but is more discussed as protozoa. *Microsporidia* has small, resistant spores; the phylum consists of 170 genera and 1300 species [39].

The lifecycle of *Microsporidia* consists of three phases, termed infective, proliferative, and sporogony [39,40]. *Microsporidia* can be transmitted through urine, soil, water, and food [41].

*Microsporidia* infection may affect muscles, intestines, gall bladder, liver, kidneys, eyes, brain, lungs, skin, and the nasal sinuses. Bowel *Microsporidia* most commonly occur in 30–50% of AIDS patients with chronic diarrhea [40].

The diagnosis of *Microsporidia* is determined by the use of several methods such as Giemsa, Gram, and/or Ziehl–Neelsen staining, to find spores containing polar filaments [39]. Touch preps and smears from biopsy, eye scraping, tissue specimens, and aspirations stained with Gram, Giemsa, or Trichrome can be identified in urine, stool, and duodenal fluid samples [39].

There are variations in *Microsporidia*, which help identify genus and species; they include the number of spores produced in sporogony, the way spores are produced, host interactions with parasites, morphological characteristics of developmental stages, nucleation, the place where infection occurs, and serological and molecular diagnosis [40].

Molecular examination methods for diagnosing *Microsporidia* include fluorescence, electron microscopy, ELISA, and Western blotting [39]. Immunofluorescence tests using the chitin binding Fluorochrome Uvitex 2B, Fungifluor, Calcofluor white, and Fungiqual A are available for detecting microsporidial infection. Monoplex PCR can also be used to detect *Microsporidia* [22].

### I. belli

*Cystoisospora belli* is a synonym for *I. belli*, which belongs to the family Eimeridae, consisting of 15 species. *Cystoisospora* or *I. belli* is only found in humans, whereas other species such as *Cystoisospora felis, Cystoisospora canis*, an*d Cystoisospora suis* are present in animals [42].

Coccidian parasites (*Cryptosporidium* spp., *I. belli*, and *Cyclospora* spp.) are the most common enteric parasites in immunocompromised patients, which can cause severe, deadly diarrhea [33]. Of the eight chronic cases of isosporiasis persisted with standard antimicrobial therapy and secondary prophylaxis, four patients died, two remained symptomatic, and two recovered. Mortality from *I. belli* infection in HIV/AIDS is high, especially at low CD4 + levels [43].

**Figure-3:**
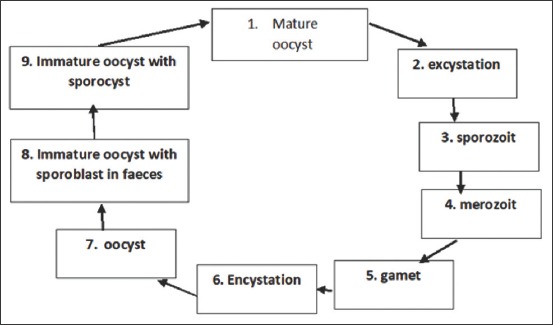
Life cycle of *Isospora belli*.

Human-to-human transmission is impossible in *I. belli* infection as the formation of an infective stage requires an external environment. The lifecycle of *I. belli* (Figure-3) begins when oocysts released in the non-infective *I. belli* become infective after being outside the body or in the environment. *I. belli* is not a zoonotic disease because humans are the only definitive host; there is no intermediate host. Transmission occurs through anal-oral contact, contaminated water, and food [44].

The method of the examination for diagnosing *I. belli* is the same as that for *C. parvum* [44]. The treatment for *I. Belli* infection is TMP/SMX regiment [44]. The low prevalence and variations of isosporiasis could be due to the wide use of anti-opportunistic infection medications, ART, and geographic differences. Furthermore, it has been suggested that co-trimoxazole prophylaxis given for other infections in AIDS cases, and the low number of oocysts excreted is possible explanations for the observed low prevalence of isosporiasis [18].

### Sarcocystis

HIV infection has altered both the epidemiology and outcome of enteric opportunistic parasitic infections. Astudy in Iran showed that the most common opportunistic and non-opportunistic pathogens were *C. parvum*, followed by *E. bieneusi*, *G. lamblia*, *Sarcocystis* spp., and *B. hominis* [41]. Research in Malaysia also showed that *Sarcocystis* is a potentially infectious disease in travelers [45]. Infection with intestinal sarcocystosis causes a self-limiting disease characterized by nausea, abdominal pain, and diarrhea in immunocompetent persons whereas in HIV/AIDS patients it could cause myopathy [46].

*Sarcocystis* was first discovered in pork meat. In the beginning, it was named sarcosporidium as the culture media produced hyphae and mycelium before clearer structure could be seen microscopically. *Sarcocystis* species infection is rare [45].

The morphology of *Sarcocystis* oocyst resembles that of *I. belli*, while the sporocysts are larger than those of *C. parvum* [42]. Intermediate hosts of *Sarcocystis* are herbivores, mammals, reptiles, birds, fish, and humans. The definitive host contains intestinal stage parasites, which vary from carnivorous animals to humans [44]. Transmission of *Sarcocystis* infection occurs from the consumption of raw meat containing the sarcocysts or ingesting water or food contaminated with the oocysts [44].

*Sarcocystis hominis* is found in cattle, while *S. suihominis* is found in pigs [44,45]. The clinical manifestation of the infection is generally asymptomatic; symptoms may involve diarrhea, abdominal pain, and weight loss [44]. Other symptoms include fever, headache, myalgia, and myositis in severe conditions caused by the development of intramuscular cysts. Clinical symptoms caused by *S. suihominis* are more severe than those of *S. hominis*. Presumptive diagnosis for *Sarcocystis* is done by taking into account any history of recent travel to tropical areas, increased serum enzymes, and eosinophilia [45].

The method of the diagnosis for *Sarcocystis* is similar to that for *C. parvum* and *I. belli*; muscle biopsy needs to be performed when myalgia is found [44]. The treatment of *S. suihominis* and *S. hominis* infections is with SMX-TMT, otherwise symptoms in the muscles are essentially treated with anti-inflammatory drugs [45].

### B. hominis

*B. hominis* is a eukaryotic, cosmopolitan parasite, infecting various animals ranging from birds, pigs, horses, amphibians, reptiles, to insects [47]. *B. hominis*, which is commonly found in the intestinal tract, can lead to gastrointestinal symptoms such as diarrhea, nausea, abdominal pain, vomiting, and bloating. The prevalence of *B. hominis* is 1.5-10% in developed countries, where it can cause deterioration of health in immune-compromised patients. *B. hominis* can be harmful to HIV-positive patients or organ-transplant patients who receive immunosuppressive agents [48].

The morphology of *B. hominis* varies between the cystic, vacuolar, multi-vacuolar, granular, and amoeboid forms. The pathognomonic characteristic of *B. hominis* is the large centralized vacuoles [47]. The protozoa are commonly found in individual feces without any symptoms, which show this parasite is of low pathogenicity [47].

*B. hominis* infects the cecum and colon. The prevalence of *B. hominis* ranges from 1.6 to 50%. The prevalence of *B. hominis* infection was 72% in people with HIV/AIDS in Indonesia [47]. Research in the United States and Paris showed that *B. hominis* infections were more frequent compared to *Entamoeba histolytica*, *G. lamblia*, and *C. parvum*in HIV/AIDS patients [49].

The lifecycle of *B. hominis* starts from the solid-walled cysts in feces, infects the host to proceed to asexual multiplication, becomes vacuolar form, arranges a multi-vacuolar or amoeboid form, and becomes a pre-cyst. Further development of the pre-cyst can be a thick-or thin-walled cyst. Thin-walled cysts play a role in the auto-infection process, while thick-walled ones will be released in the stool and are the infective form later in fecal-oral or external transmission [47,49].

Preferred therapies for *B. hominis* infections are emetin, furazolidone, metronidazole, and TMP-SMX. Metronidazole at a dose of 250-750mg 3times a day for 5days or 2g/day for 5days is a therapeutic regimen to treat *B. hominis* infection in immunocompetent patients with symptoms of diarrhea, abdominal pain, and nausea [47]. In HIV/AIDS patients, treatment with furoximin becomes the choice to eradicate *B. hominis* [47]. Another study using a 0.5-1g metronidazole regimen for 7-10days, showed relieve of symptoms accompanied by a diminished concentration of *B. hominis* in feces [47].

### C. neoformans

*C. neoformans* is a fungus, which is commonly found in urban areas. It may infect immunocompromised individuals who ingest the spores. In individuals with adequate immune systems, *C. neoformans* vars gatti is generally asymptomatic, however, in immunocompromised individuals, it becomes a major cause of fatal neurological symptoms, such as meningitis [50]. Risk factors for *C*. *neoformans* infection include patients undergoing organ transplants and other long-term immunosuppressive therapy [50,51]. Capsules, melanin, and urease phospholipase activity are the virulence factors of *C. neoformans*, which play an important role in environmental survival [51,52]. *C. neoformans* can cause chronic diarrhea in non-HIV infected patients. Chronic diarrhea caused by *C. neoformans* is less frequent, and clinical suspicion is required to determine diagnosis [53].

The main treatment for infection caused by *C. neoformans* is amphotericin as a secondary metabolite of *Streptomyces nodosus*. For the treatment of *C. neoformans* infection in HIV/AIDS patients, HIV protease inhibitor such as indinavir may be used [50].

## Diagnosis and Supporting Examination for Detecting Diarrhea-causing Parasites in HIV/AIDS Patients

The examination methods for diagnosing intestinal parasitic infections in patients with HIV are selected based on the organism. The methods include formalin-ethyl concentration technique for protozoa and worms, modified acid-fast staining for coccidian that cause classical opportunistic infections (*C. parvum*, *I. belli*, *C. cayetanensis*, and *S. hominis*), whereas microspora examination is done with Trichrome staining [17,18]. Stool samples were examined in iodine preparation for the detection of protozoan oocysts and cysts. Permanent stained smears are performed with modified Ziehl–Neelsen staining (Cold Method) for the detection of oocysts of *Cryptosporidium, Isospora, and Cyclospora* [10]. Simple direct, concentrated, and stained smear examination of stool can help in the identification of enteric parasites in majority of patients [7].

## Diagnostic Molecular Methods for Opportunistic Parasitic Infections

After the discovery of nucleic acid-based detection, the multiplex real-time PCR method has been applied in diagnosing intestinal parasites in the Netherlands. The advantage of this method of detection and quantification of parasite-specific DNA are that it is very sensitive, thus increases detection of intestinal parasites, namely, *Giardia lamblia* and *Cryptosporidium* spp. [54]. Monoplex PCR is used to detect *Microsporidia* and *Cryptosporidium*, whereas multiplex PCR is used for *G. lamblia*, *Cryptosporidium*, *Dientamoeba fragilis*, and *E. histolytica* [39]

Molecular diagnostic methods for *C. parvum* include lateral flow immunoassays, immunochromatographic assays, and direct fluorescent antibody tests. ATaqMan PCR assay targeting, the 18S ribosomal DNA (rDNA) has been found to detect *Cryptosporidium* species [22]. Molecular diagnostic methods for *C. cayetanensis* include autofluorescence under UV, epifluorescence microscopy, and DNA amplification using PCR. Areal-time PCR targeting, the internal transcribed spacer two regions of the rRNA gene have also been used to examine *I. belli* DNA in fecal samples [22].

The PCR method is useful for determining *Microsporidia* species because there are 14 *Microsporidia* species. In addition to the PCR method, the immunofluorescent-antibody test has the same sensitivity as PCR [22]. *Sarcocystis* can be detected using transmission electron microscopy [55]. The method of PCR-restriction fragment length polymorphism was used to amplify approximately 900bp fragment at the 18S rRNA (SSU) gene, of which the restriction enzyme BclI was used for the identification of *Sarcocystis* species in Iran [56].

The diagnosis of *B. hominis* can be made using the methods of conventional, phase-contrast, and electron microscopies, cultivation, sero-diagnosis, and using molecular methods [57]. *Blastocystis* exhibits extensive genetic diversity, and this has been documented using numerous molecular techniques [22]. PCR was performed using seven primer pairs targeting the SSU rDNA gene, and sequencing have been used to detect *B. hominis* in Iran. Multiplex PCR using two primer sets were tested (CN4-CN5) and the multiplex CNa70S-CNa70A/CNb49S-CNb-49A that amplified a specific product for *C. neoformans* and another for *C. gattii* are [58] already established [59].

Molecular diagnosis methods for the identification of *Cyclospora* oocysts are hampered by the lack of animal models and limited DNA sequence data. Techniques for fingerprint analysis and genotype discrimination are not available for *C. cayetanensis*; therefore, identification using a microscope is still frequently used [21]. *Microsporidia* are difficult to detect because of their small size, their slowly infecting properties, and sometimes asymptomatic infection. Immunoblot, ELISA, and IFA using monoclonal and polyclonal antibodies also help serological examination for *Microsporidia* characterization. However, serological testing cannot be relied on in people with HIV because of lack of immunity [23].

Simple India ink examination of the CSF has a 70-90% sensitivity and cases that are negative on the India ink test, can be reliably diagnosed by detection of cryptococcal antigen. Antigen detection based on latex agglutination although sensitive and specific were never widely available in high-burden, resource-limited settings. In the context of limited resources, development of a lateral flow assay (LFA; manufactured by IMMY) has been a major advancement. Asecond, semi-quantitative, lateral flow test is now in development by Biosynex and Institut Pasteur in Paris, France [60].

The association between intestinal parasitic infections and HIV infection has been well documented in many research [2,4,7,10,13,14,16,18,22,31,34,46,61]. The prevalence of intestinal parasitic infections was varied depending on geographic and other factors such as CD4+ count, ART, prophylaxis, hygiene, and sanitation (Table-2) [1,4,7,9,11,12,14,19,33]. Different examination methods that have been used to investigate intestinal parasitic infections also influenced the prevalence of the diseases. Various molecular diagnosis methods have been used for detection of opportunistic parasitic infections (Table-3) [22,23,39,54,55,56,57,58,59].

**Table-2:** Prevalence, risk factors, and examination methods of opportunistic parasitic infection in HIV/AIDS patients based on geographical regions.

Prevalence of intestinal parasitic infection	Risk factors	Examination methods	References	Published year	Country
30.6%	CD 4 count<200 cell/μl	Wet mount, formol–ether sedimentation and modified Ziehl–Neelsen techniques	[19]	2015	Ethiopia
32.5%	CD 4 count<200 cell/μl	Direct wet-mounts (normal saline, Lugol’s iodine), formol-ether concentration, modified Ziehl–Neelsen, modified trichrome staining	[11]	2015	India
45%	CD 4 count<200 cell/μl	Direct microscopy by saline wet mount, iodine wet mount, modified Ziehl–Neelsen (ZN)	[7]	2016	India
45%	CD 4 count 200-500 cell/ μl, Health education regarding personal hygiene, regular de-worming	Direct wet mount, formalin ether concentration, modified Ziehl–Neelsen (ZN)	[33]	2016	Laos
49%	Age group 20-30 years, geographis, personal hygiene, sanitary habits, method of stool examination, immune status	Iodine, saline, formalin ether concentration	[4]	2016	India
pre ART 84.6%, ART 82.3%	CD 4 count<200 cell/μl	Wet mount, Iodine mount, Katokatz, formalin ether concentration, modified Ziehl–Neelsen (ZN), Modified field staining	[1]	2016	Cameroon
85%	CD 4 count<200 cell/μl	Direct wet mount (saline and Lugol’s Iodine), formalin ether concentration, modified Ziehl–Neelsen (ZN), Trichrome staining, Iron Hematoxylin staining	[12]	2016	India
28.18%	CD 4 count<500 cell/μl, Presence of domestic animals, poor sanitation	Direct wet mount, formol-ether sedimentation modified Ziehl-Neelsen	[14]	2018	Ethiopia
26.4%	CD 4 count<200 cell/μl, ART, Trimethoprim-sulfamethoxazole (TS) prophylaxis	Direct wet mount, Ritchie and modified Ziehl–Neelsen techniques, Coproantigen	[9]	2017	Mozambique

ART=Antiretroviral therapy, HIV=Human immunodeficiency virus, AIDS=Acquired immunodeficiency syndrome

**Table-3:** Diagnosis methods based on modern molecular biology of opportunistic parasitic infection.

Parasite species	Molecular biology based methods of diagnosis	References
*Cryptosporidium parvum*	Monoplex PCR	[39]
	Multiplex PCR	[54]
	Lateral Flow Immunoassay, Immunochromatographic assay, Direct Fluorescent Antibody Test, TaqMan PCR	[22]
*Cyclospora cayetanensis*	Autofluorescent under UV EpiFluorescent, PCR	[22]
*Isospora belli*	PCR, Real time PCR	[22]
*Blastocyst hominis*	Phase contrast electron microscopy, serodiagnosis, PCR	[57,59]
*Sarcocystis*	Transmission Electron Microscopy	[55]
	PCR-RFLP	[56]
*Microsporidia*	Immunofluorescent Test, IFAT	[22]
	Monoplex PCR	[39]
	Immnuoblot, ELISA, IFAT	[23]
*Cryptococcus neoformans*	Multiplex PCR	[58]

PCR=Polymerase chain reaction, RFLP=Restriction fragment length polymorphism, IFAT=Immunofluorescence antibody test

## Treatment for Opportunistic Parasitic Infections

The treatment for *B. hominis* is metronidazole [57]. The drugs of choice for cryptosporidiosis include paromomycin, azithromycin, and nitazoxanide [22]. Effective treatment for *C. cayetanensis* infection is TMP-SMX; the alternative therapy is nitazoxanide, or ciprofloxacin. Treatment for *I. belli* infection is co-trimoxazole for 7-10days [22]. Albendazole has been shown to be effective in treating infections caused by *Microsporidia*. Other drugs of choice for *Microsporida* include nitazoxanide and furazolidone [22]. Amphotericin B plus flucytosine rapid clearance is used for *C. neoformans* [60].

## Conclusion and Future Perspective

Opportunistic parasitic infections are the most common health problems in patients with HIV/AIDS. Opportunistic intestinal parasitic infections will increase in the future due to the increasing incidence of HIV/AIDS in the population. However, early detection, using molecular diagnosis methods, of intestinal parasitic infections in HIV seropositive patients, appropriate initial treatment in new cases, and maintenance therapy in chronic cases will improve the quality of life of people infected with HIV.

## Authors’ Contributions

DAL had the idea, compiled, managed team and source, also wrote the manuscript, LTS, MM, and MS provided journal content, and prepared the manuscript. KS drafted and finalized the manuscript. All authors read and approved the final manuscript.

## References

[1] Al-Qobati S.A, Al-Nabehi B.A, Mohamad A.A, Al-Nabbhi A.S, Al-Kadi M.A (2018). Enteric protozoal infections among immunocompromised and immunocompetent people, Sana'a Town, Yemen. EC Microbiol.

[2] Cerveja B.Z, Tucuzo R.M, Madureira A.C, Nhacupe N, Langa I.A, Buene T, Banze L, Funzamo C, Noormahomed E.N (2017). Prevalence of intestinal parasites among HIV infected and HIV uninfected patients treated at the 1°De Maio health centre in Maputo, Mozambique. EC Microbiol.

[3] Yoshimura K (2017). Current status of HIV/AIDS in the ART era. J. Infect. Chemother.

[4] Nsagha D.S, Njunda A.L, Assob N.J.C, Ayima C.W, Tanue E.A, Kibu O.D, Kwenti T.E (2016). Intestinal parasitic infections in relation to CD4^+^T cell counts and diarrhea in HIV/AIDS patients with or without antiretroviral therapy in Cameroon. BMC Infect. Dis.

[5] Shenoy N, Ramapuram J.T, Shenoy A, Ahmed J, Srikant N (2017). Incidence of opportunistic infections among HIV-positive adults on highly active antiretroviral therapy a teaching hospital, India:Prospective study. J. Int. Assoc. Provid. AIDS Care.

[6] Patel S.D, Javadekar T.B, Kinariwala D.P (2015). Enteric opportunistic parasitic infections in HIV seropositive patients at tertiary care teaching hospital. Natl. J. Med. Res.

[7] Rao R.P (2016). Study of opportunistic intestinal parasitic infections in HIV seropositive patients at a tertiary care teaching hospital in Karnataka, India. Int. J. Contem. Med. Res.

[8] Agarwal S.G, Powar R.M, Tankhiwale S, Rukadikar A (2015). Study of opportunistic infections in HIV-AIDS patients and their co-relation with CD4+cell count. Int. J. Curr. Microbiol. Appl. Sci.

[9] Dyab A.K, Gaber M.A, Hassan T.M, El Kady A.M, Badary D.M, Mahmoud H.S (2016). Parasites associated with human immune-deficiency virus (HIV) infection in Assiut university hospitals, Egypt. Madridge J. Vaccines.

[10] Surekha Y.A, Shilpa H.S, Jeer M, Krishna S (2016). Intestinal parasites in HIV infected individuals and its correlation with the CD4 counts. Int. J. Curr. Microbiol. Appl. Sci.

[11] UNAIDS (2008). Report on the Global AIDS Epidemic.

[12] Nissapatorn V, Sawangjaroen N (2011). Parasitic infections in HIV infected individuals :Diagnostic and therapeutic challenges. Indian J. Med. Res.

[13] Khalil P.S, Mirdha B.R, Sinha S, Panda S, Singh Y, Joseph A, Deb M (2015). Intestinal parasitosis in relation to anti-retroviral therapy, CD4+T-cell count and diarrhea in HIV. Korean J. Parasitol.

[14] Shilpa H.S, Mariraj J (2016). Intestinal parasitic infections in relation to HIV/AIDS status, diarrhoea and CD4 T-cell count. Int. J. Curr. Microbiol. Appl. Sci.

[15] Vaillant A.A, Naik R (2020). HIV-1 Associated Opportunistic Infections.

[16] Udeh E.O, Obiezue R, Okafor F.C, Ikele C.B, Okoye I.C, Otuu C.A (2019). Gastrointestinal parasitic infections and immunological status of HIV/AIDS coinfected individuals in Nigeria. Ann. Glob. Health.

[17] Paboriboune P, Phoumindr N, Borel E, Sourinphoumy K, Phaxayaseng S, Luangkhot E, Sengphilom B, Vansilalom Y, Odermatt P, Delaporte E, Etard J.F (2014). Intestinal parasitic infections in HIV-infected patients, lao people's Democratic republic. PLoS One.

[18] Kiros H, Nibret E, Munshea A, Kerisew B, Adal M (2015). Prevalence of intestinal protozoan infections among individuals living with HIV/AIDS at Felegehiwot referral hospital, Bahir Dar, Ethiopia. Int. J. Infect. Dis.

[19] Solomon F.B, Angore B.N, Koyra H.C, Tufa E.G, Berheto T.M, Admasu M (2018). Spectrum of opportunistic infections and associated factors among people living with HIV/AIDS in the era of highly active anti-retroviral treatment in Dawro zone hospital:A retrospective study. BMC Res Notes.

[20] Kindie Y, Bekele S (2016). Prevalence and risk factors for intestinal parasite infections in HIV/AIDS patients with anti-retroviral treatment in South West Ethiopia. J. Trop. Dis.

[21] Chacin-Bonilla L, Rose J.B, Jiménez-Cisneros B (2017). Cyclospora cayetanensis. Global Water Pathogens Project.

[22] Stark D, Barrat J.L, Van Hal S, Marriot D, Harkness J, Ellis J.T (2009). Clinical significance of enteric *Protozoa* in the immunosuppressed human population. Clin. Microbiol. Rev.

[23] Faisal A.F, Bokhari A.A (2020). Microsporidium.

[24] Aranzales A, Radon K, Froeschl G, Pinzon A, Delius M (2018). Prevalence and risk factors for intestinal parasitic infections in pregnant women residing in three districts of Bogotá, Colombia. BMC Public Health.

[25] Aneth M, Johnson M (2016). Prevalence of parasitic infections and associations with pregnancy complications and outcomes in Northern Tanzania:A registry-based cross-sectional study. BMC Infect. Dis.

[26] Pumipuntu N, Piratae S (2018). Cryptosporidiosis:A zoonotic disease concern. Vet. World.

[27] Sanchez R (2010). Update on *Cyclospora cayetanensis* a food-borne and waterborne parasite. Clin. Microbiol. Rev.

[28] Alfano-sobsey E.M, Eberhard M.L, Seed J.R, Weber D.J, Won K.Y, Nace E.K, Moe C.L (2004). Human challenge pilot study. Emerg. Infect. Dis.

[29] Robertson L.J, Gjerde B, Campbell A.T (2006). Isolation of *Cyclospora* oocysts from fruits and vegetables using lectin-coated paramagnetic beads. J. Food Prot.

[30] Alemu G, Alelign D, Abossie A (2018). Prevalence of opportunistic intestinal parasites and associated factors among HIV patients while receiving ART at Arba Minch hospital in Southern Ethiopia:A cross-sectional study. Ethiop. J. Health Sci.

[31] Liu S, Roellig D.M, Guo Y, Li N, Frace M.A, Tang K, Zhang I, Feng Y, Xiao I (2016). Evolution of mitosome metabolism invasion-related protein in *Cryptosporidium*. BMC Genomic.

[32] Mohaghegh M.A, Hejazi S.H, Ghomashlooyan M, Kalani H, Mirzaei F, Azami M (2017). Prevalence and clinical features of *Cryptosporidium* infection in hemodialysis patients. Gastroenterol. Hepatol. Bed Bench.

[33] Salehi S.G, Mirjalali H, Farnia S, Rezaeian M (2016). Prevalence of intestinal coccidial infections among different groups of immunocompromised patients. Iran. J. Parasitol.

[34] Tandel J, English E.D, Sateriale A, Gullicksrud J.A, Beiting D.P, Sullivan M.C, Pinkston B, Striepen B (2019). Life cycle progression and sexual development of the apicomplexan parasite *Cryptosporidium parvum*. Nat. Microbiol.

[35] Shrivastava A.K, Kumar S, Smith W.A, Sahu P.S (2017). Revisiting the global problem of cryptosporidiosis and recommendations. Trop. Parasitol.

[36] Khurana S, Chaudhary P (2018). Laboratory diagnosis of cryptosporidiosis. Trop. Parasitol.

[37] Haserick J.R, Klein J.A, Costello C.E, Samuelson J (2017). *Cryptosporidium parvum* vaccine candidates are incompletely modified with O-linked-N-acetylgalactosamine or contain N-terminal N-myristate and S-palmitate. PLoS One.

[38] Bangoh B, Voskuijl J, Thitiri W, Menting S, Verhaar N, Mwalekwa L, de Jong D.B, van Loenen M, Mens P.F, Berkley J.A, Bandsma R.H.J, Schallig H.D (2019). Performance of three rapid diagnostic tests for the detection of *Cryptosporidium* spp and *Giardia duodenalis* in children with severe acute malnutrition and diarrhoea. Infect. Dis. Poverty.

[39] Garcia L.S (2002). Minireview laboratory identification of the *Microsporidia*. J. Clin. Microbiol.

[40] Cali A, Takvorian P.M (2011). Microsporidiosis. In:Topics on the Pathology of Protozoan and Invasive Arthropod Diseases.

[41] Agholi M (2013). HIV/AIDS-associated opportunistic protozoal diarrhea. AIDS Res. Hum. Retrovirus.

[42] Rodriguez-morales A.J (2014). *Protozoa*
*Cystoisospora belli*(Syn *Isospora belli*). Encyclopedia of Food Safety. Vol. 2.

[43] Tom B, John B, Graeme M, Marc M (2012). Failure to eradicate *Isospora belli* diarrhoea despite immune reconstitution in adults with HIV a case series. PLoS One.

[44] Heelan J.S, Frances W.I (2002). Essentials of Human Parasitology.

[45] Fayer R, Esposito D.H, Dubey P (2015). Human infections with sarcocystis species. Clin. Microbiol. Rev.

[46] Anderson D, Nathoo N, Lu J.Q, Kowalewska-Grochowska K.T, Power C (2018). Sarcocystis myopathy in a patient with HIV-AIDS. J. Neurovirol.

[47] Sadaf H.S, Khan S.S, Urooj K.S, Asma B, Ajmal S.M (2013). *Blastocystis hominis*-potential diahorreal agent:A review. Int. Res. J. Pharm.

[48] Chen C.H, Sun H.Y, Chien H.F, Lai H.S, Chou N.K (2014). *Blastocystis hominis* infection in a post-cardiotomy patient on extracorporeal membrane oxygenation support:A case report and literature review. Int. J. Surg. Case Rep.

[49] Wawrzyniak I, Poirier P, Viscogliosi E, Dionigia M, Texier C, Delbac F, Alaoui H.E (2013). *Blastocystis* an unrecognized parasite :An overview of pathogenesis and diagnosis. Ther. Adv. Infect. Dis.

[50] Qadri S.M.H, Al-Okaili G.A, Al-Dayel F (1989). Clinical significance of *Blastocystis hominis*. J. Clin. Microbiol.

[51] Mcclelland E.E, Casadevall A, Eisenman H.C (2007). Pathogenesis of *Cryptococcus neoformans* pathogenesis of *Cryptococcus neoformans*. New Insights in Medical Mycology.

[52] Kwon-Chung K.J, Fraser J.A, Doering T.L, Wang Z, Janbon G (2014). *Cryptococcus neoformans* and *Cryptococcus gattii* the etiologic agents of Cryptococcosis. Cold Spring Harb. Perspect. Med.

[53] Sungkanuparph S, Tanphaichitra D, Pracharktam R (2003). Chronic diarrhea caused by *Cryptococcus neoformans* in a non-human immunodeficiency virus-infected patient. Scand. J. Infect. Dis.

[54] Lieshout L, Roestenberg M (2015). Clinical consequences of new diagnostic tool for intestinal parasites. Clin. Microbiol. Infect.

[55] Choi T.I, Hong E.J, Ryu S.Y, Sim C, Chae J.S, Kim H.C, Park J, Choi K.S, Yu D.H, Yoo J.G, Park B.K (2018). Detection and identification of *Sarcocystis cruzi*(*Protozoa*
*Apicomplexa*) by molecular and ultrastructural studies in naturally infected Korean cattle (*Bos taurus coreanae*) from Daejeon, Korea. Korean J. Parasitol.

[56] Hooshyar H, Abbaszadeh Z, Sharafati-Chaleshtori R, Arbabi M (2017). Molecular identification of *Sarcocystis* species in raw hamburgers using PCR-RFLP method in Kashan, central Iran. J. Parasit. Dis.

[57] Natasa T, Milenković M, Vera B, Dragan Z, Aleksandar T (2017). *Blastocystis hominis*:A mysterious and commonly disregarded parasite. Facta Univ. Ser. Med. Biol.

[58] Martin A, Brighente K.B, Matos T.A, Vidal J.E, Hipólito D.D, Pereira-Chioccola V.L (2015). Molecular diagnosis of cryptococcal meningitis in cerebrospinal fluid:Comparison of primer sets for *Cryptococcus neoformans* and *Cryptococcus gattii* species complex. Braz. J. Infect. Dis.

[59] Khademvatan S, Masjedizadeh R, Razin E.Y, Mahbodfar H, Rahim F, Yousefi E, Foroutan M (2018). PCR-based molecular characterization of *Blastocystis hominis* subtypes in Southwest of Iran. J. Infect. Public Health.

[60] Williamson P, Jarvis J.N, Panackal A, Fisher M.C, Molloy S.F, Loyse A, Harrison T (2016). Cryptococcal meningitis:Epidemiology, immunology, diagnosis and therapy. Nat. Rev. Neurol.

[61] Piranshahi A.R, Tavalla M, Khademvatan S (2018). Genomic analysis of *Blastocystis hominis* isolates in patients with HIV-positive using locus SSU-rDNA. J. Parasit. Dis.

